# Phosphorylation at Serines 157 and 161 Is Necessary for Preserving Cardiac Expression Level and Functions of Sarcomeric Z-Disc Protein Telethonin

**DOI:** 10.3389/fphys.2021.732020

**Published:** 2021-09-08

**Authors:** Hannah R. Lewis, Seda Eminaga, Mathias Gautel, Metin Avkiran

**Affiliations:** ^1^School of Cardiovascular Medicine and Sciences, St Thomas’ Hospital, King’s College London British Heart Foundation Centre of Research Excellence, London, United Kingdom; ^2^School of Basic and Medical Biosciences, Guy’s Hospital, King’s College London British Heart Foundation Centre of Research Excellence, London, United Kingdom

**Keywords:** telethonin, TCAP, cardiac hypertrophy, phosphorylation, proteasome

## Abstract

**Aims:** In cardiac myocytes, the sarcomeric Z-disc protein telethonin is constitutively bis-phosphorylated at C-terminal residues S157 and S161; however, the functional significance of this phosphorylation is not known. We sought to assess the significance of telethonin phosphorylation *in vivo*, using a novel knock-in (KI) mouse model generated to express non-phosphorylatable telethonin (*Tcap*^S157/161A^).

**Methods and Results:***Tcap*^S157/161A^ and wild-type (WT) littermates were characterized by echocardiography at baseline and after sustained β-adrenergic stimulation *via* isoprenaline infusion. Heart tissues were collected for gravimetric, biochemical, and histological analyses. At baseline, *Tcap*^S157/161A^ mice did not show any variances in cardiac structure or function compared with WT littermates and mutant telethonin remained localized to the Z-disc. Ablation of telethonin phosphorylation sites resulted in a gene-dosage dependent decrease in the cardiac telethonin protein expression level in mice carrying the S157/161A alleles, without any alteration in telethonin mRNA levels. The proteasome inhibitor MG132 significantly increased the expression level of S157/161A telethonin protein in myocytes from *Tcap*^S157/161A^ mice, but not telethonin protein in myocytes from WT mice, indicating a role for the ubiquitin–proteasome system in the regulation of telethonin protein expression level. *Tcap*^S157/161A^ mice challenged with sustained β-adrenergic stimulation *via* isoprenaline infusion developed cardiac hypertrophy accompanied by mild systolic dysfunction. Furthermore, the telethonin protein expression level was significantly increased in WT mice following isoprenaline stimulation but this response was blunted in *Tcap*^S157/161A^ mice.

**Conclusion:** Overall, these data reveal that telethonin protein turnover *in vivo* is regulated in a novel phosphorylation-dependent manner and suggest that C-terminal phosphorylation may protect telethonin against proteasomal degradation and preserve cardiac function during hemodynamic stress. Given that human telethonin C-terminal mutations have been associated with cardiac and skeletal myopathies, further research on their potential impact on phosphorylation-dependent regulation of telethonin protein expression could provide valuable mechanistic insight into those myopathies.

## Introduction

Telethonin, also known as titin-cap (T-cap) protein, is expressed in sarcomeric Z-discs of both cardiac and skeletal myocytes (Valle et al., 1997; Mason et al., 1999), where the protein’s N-terminal domain acts as a cross linker between the Z1 and Z2 domains of two antiparallel titin molecules (Zou et al., 2003, 2006). This super-stable interaction is mediated through a β-sheet complex and is critical for the targeting of telethonin to the Z-disc (Pinotsis et al., 2006; Zou et al., 2006). Telethonin has also been identified as a putative binding partner for several other proteins, including muscle LIM protein (MLP), myostatin and the β-subunit of the delayed rectifier K^+^ channel, MinK (Furukawa et al., 2001; Knoll et al., 2002; Nicholas et al., 2002). However, the physiological significance of these interactions remains to be determined. Human telethonin mutations have been associated with both hypertrophic and dilated cardiomyopathies, as well as the skeletal myopathy limb-girdle muscular dystrophy type 2G (LGMD2G) (Moreira et al., 2000; Hayashi et al., 2004). Telethonin knock-out (KO) in mice, surprisingly, has been reported to induce no changes in muscle function or Z-disc architecture in skeletal and cardiac tissues at baseline (Markert et al., 2010; Knoll et al., 2011). Nevertheless, in response to mechanical stress (chronic pressure overload), KO mice develop maladaptive cardiac hypertrophy and display abnormal cardiac function, compared to wild-type (WT) controls, accompanied by cardiac fibrosis and apoptosis (Knoll et al., 2011). Furthermore, cardiomyocytes isolated from KO mice have been reported to display significant alterations in Ca^2+^ transient dynamics and the structure of the t-tubule network (Ibrahim et al., 2013).

Telethonin function appears to be regulated by post-translational modification, with two C-terminal phosphorylation sites identified at serine residues 157 and 161 (S157 and S161) (Mayans et al., 1998; Pinotsis et al., 2006; Candasamy et al., 2014). These two phospho-acceptor sites are targeted by protein kinase D (PKD), Ca^2+^/calmodulin-dependent kinase II (CaMKII) and possibly by titin kinase *in vitro* (Mayans et al., 1998; Candasamy et al., 2014). Furthermore, previous work from our group has shown that in both isolated cardiomyocytes and cardiac ventricular tissue, telethonin is detected in a predominantly bis-phosphorylated form, with only small proportions of the protein present in a mono- and non-phosphorylated state (Candasamy et al., 2014). We have additionally demonstrated that heterologous expression of S157/161A telethonin in isolated cardiomyocytes disturbs the kinetics of intracellular Ca^2+^ transients (Candasamy et al., 2014). Nevertheless, the functional significance of telethonin C-terminal phosphorylation is not well understood. The ubiquitin–proteasome system (UPS) may also have a role in the regulation of telethonin, which has been reported as a putative binding partner of the E3 ubiquitin-ligating enzymes MDM2, MURF1, and MURF2 (Witt et al., 2005; Tian et al., 2006). Expression of these E3 enzymes *in cellula* led to a decrease in telethonin protein expression, which was inhibited by the addition of the proteasome inhibitor MG132 (Tian et al., 2006; Polge et al., 2018). Furthermore, proteomic analysis of the murine heart identified K148 at the C-terminus of telethonin as a putative ubiquitylation site (Wagner et al., 2012). The proximity of this residue to the two C-terminal phosphorylation sites suggests that the presence or absence of telethonin phosphorylation may have a role in regulation of ubiquitylation and subsequent degradation by the UPS.

In this study, we aimed to characterize the functional significance of telethonin phosphorylation at S157 and S161. We present results from a novel knock-in (KI) mouse model, which expresses telethonin with serine-to-alanine (S/A) substitutions at those residues (S157/161A), rendering the protein non-phosphorylatable. Our results indicate that telethonin protein expression levels are subject to regulation by phosphorylation-sensitive processing *via* the UPS. We also demonstrate that phosphorylation of telethonin at S157/S161 is required for an increase in telethonin protein expression levels in response to sustained hemodynamic stress, which may be necessary for the preservation of cardiac function.

## Materials and Methods

Expanded methods can be found in Supplementary Material. The data underlying this article are available in the article and in its online Supplementary Material.

### Generation of *Tcap*^S157/161A^ KI Mice

C57BL/6N mice carrying two point mutations in the *Tcap* gene to ablate telethonin phosphorylation sites (S157A/S161A) were generated by Taconic (Cologne) (Supplementary Methods). Mice were housed in pathogen-free, individually ventilated cages with a 12 h light-dark cycle, controlled humidity and temperature (20–22°C), fed standard mouse chow, and water *ad libitum*. The study was approved by the local Ethics Review Board and animal handling and experiments were performed according to the Home Office regulations, as detailed in the Home Office Guidance on the Operation of the Animals (Scientific Procedures) Act 1986, HMSO (London), under Directive 2010/63/EU (PPL 70/8948). Animals were euthanized by cervical dislocation under isoflurane-induced anesthesia (3–4% isoflurane *via* inhalation) according to Schedule 1 United Kingdom Animals (Scientific Procedures) Act 1986.

### Echocardiography

Echocardiography was performed as described previously (Puhl et al., 2016), using the Visual Sonics Vevo^®^ 770 imaging system (Scanhead: RMV707B, 15–45 MHz, cardiac mouse). Anesthesia was maintained *via* nose-cone inhalation of 1.5–2.0% isoflurane throughout the procedure. Body temperature was monitored and maintained at 37 ± 1.5°C to sustain a heart rate of approximately 400–600 bpm (average 522 ± 52.87 bpm for baseline measurements, recorded range 399–636 bpm).

### Chronic β-Adrenergic Stress Model

Osmotic mini pumps (Model 1002, Alzet) were implanted in male and female mice at 8–9 weeks of age, as described previously (Puhl et al., 2016). Anesthesia was maintained *via* nose-cone inhalation of 3–4% isoflurane throughout the procedure. Vetergesic (buprenorphine hydrochloride; 0.03 mg/ml) was administered *via* single intraperitoneal injection during mini-pump implantation as a post-operative analgesic. Mice were randomly allocated to receive either a pump containing 0.9% saline (vehicle control) or isoprenaline hydrochloride (Sigma I5627), dissolved in 0.9% saline and delivered at a dose of 30 mg/kg/day for 14 days. Mice were imaged *via* echocardiography 1 day prior to the implantation of the mini-pump and additionally after 14 days of infusion.

### Acute β-Adrenergic Stress Model

Echocardiographic long and short axis one-dimensional M-mode projections were taken at baseline and 2–3 min after bolus intraperitoneal injections of 0.9% saline then isoprenaline hydrochloride (Sigma I5627), dissolved in 0.9% saline to deliver a dose of 0.1 mg/kg body weight. Isoprenaline injections were administered 10 min after the administration of the 0.9% saline control.

### Immunohistochemistry and Histology

Following excision, cardiac tissues were frozen in isopentane (chilled using liquid nitrogen) and 10 μm sections were cut using a −20°C cryostat (Leica). The sections were fixed in cold acetone for 5 min at −20°C, washed in PBS and blocked in 10% normal goat serum/PBS for 1 h at RT. Sections were incubated with primary antibodies for anti-telethonin (1:250, Abcam 133646) and anti-Titin-T-12 (1:10, gift from Dr. Elisabeth Ehler, KCL) overnight at 4°C, followed by fluorescently conjugated anti-mouse Cy3 (1:500) and anti-rabbit Alexa Fluor 488 (1:500, Invitrogen) secondary antibodies. The slides were mounted with ProLong Gold Antifade reagent (Invitrogen). Sections were imaged using an inverted confocal microscope (TCS SP5 system, Leica). Alternatively (ISO study), cardiac tissues were fixed in 4% paraformaldehyde at 4°C overnight before processing and embedding in paraffin wax for tissue sectioning. Five micrometers paraffin sections were stained using Alexa Fluor 488 conjugated wheat germ agglutinin (WGA; Invitrogen) to identify myocyte cell borders or Picro Sirius Red Stain Kit (Polysciences) to detect collagen deposition. The mounted sections were imaged using a confocal microscope (WGA-fluorescent slides) or a Leica DM600B microscope (Picro Sirius Red slides). Transverse-cut myocytes with centrally located nuclei were demarcated manually to calculate myocyte-cross sectional area using NIH ImageJ software. Fibrosis was quantified by using the color-threshold method using NIH ImageJ software.

### SDS-PAGE and Immunoblotting

Proteins were separated by SDS/PAGE and Phos-tag^TM^ SDS/PAGE as previously described (Kinoshita et al., 2006; Candasamy et al., 2014). For Phos-tag SDS-PAGE, samples were resolved on 10% polyacrylamide gels supplemented with 40 μmol/L phosphate affinity reagent Phos-tag^TM^ (Wako) and 80 μmol/L MnCl_2_. The following primary antibodies were used: anti-telethonin (1:1000, Abcam 133646), anti-α-actinin (1:2000, Abcam 50599), anti-troponin I (1:1000, CST 4002), anti-GAPDH (1:10,000, CST 2118), anti-calsequestrin (1:1000, ThermoFisher PA1-913), and anti-MyBPC (1:90000) (Gautel et al., 1995). HRP-conjugated secondary antibodies anti-rabbit IgG (1:2000, CST, 7074) and anti-rat IgG (1:2000, CST 7077) were used and protein bands were visualized by ECL Western Blotting Detection Reagents (GE Healthcare) on chemiluminescence film. Protein expression was quantified by GS-800 calibrated imaging densitometer (BioRad).

### Quantitative-PCR

Quantitative-PCR (q-PCR) analysis of *Tcap*, *Pnmt*, *Col1a1*, *Col3a1*, *p53*, and *p21* was performed using Power SYBR Green PCR Master mix (ThermoFisher) according to manufacturer’s instructions. Five nanograms cDNA/reaction was analyzed in triplicate to quantify gene expression using 7900HT thermal cycler (Applied Biosystems) with the following cycling conditions: 95°C for 10 min, 40 cycles of 95°C for 1 min, 60°C for 15 s. Primer sequences were obtained using PrimerBank^1^ and are provided in Supplementary Table 1. Relative quantity was calculated using the 2^–ΔΔ*CT*^ method, using *Hprt* or *Gapdh* as reference genes.

### Treatment of Murine Cardiomyocytes With MG132

Cardiomyocytes were isolated from hearts of adult male mice by collagenase-based digestion, as described previously (Candasamy et al., 2014), and maintained in M119 culture medium supplemented with 2 mM L-creatine, 5 mM carnitine, 5 mM taurine, and 100 IU/ml penicillin/streptomycin. One hour post plating, myocytes were incubated with modified M199 culture medium containing 10 μmol/L MG132 (Sigma M7449) or vehicle control (DMSO). Cells were treated for 16 h prior to lysis in sample buffer.

### Statistical Analysis

Quantitative data are presented as mean ± SD or SE, as indicated. Statistical significance was assessed using Graph-Pad Prism 7.0 software. Data sets were analyzed by unpaired *T*-test or one or two-way ANOVA, followed by Tukey’s or Sidak’s *post hoc* test, as appropriate. Differences were considered significant when *p* < 0.05.

## Results

### Basal Cardiac Phenotype of Mice With Ablation of Telethonin Phosphorylation Sites

**FIGURE 1:**
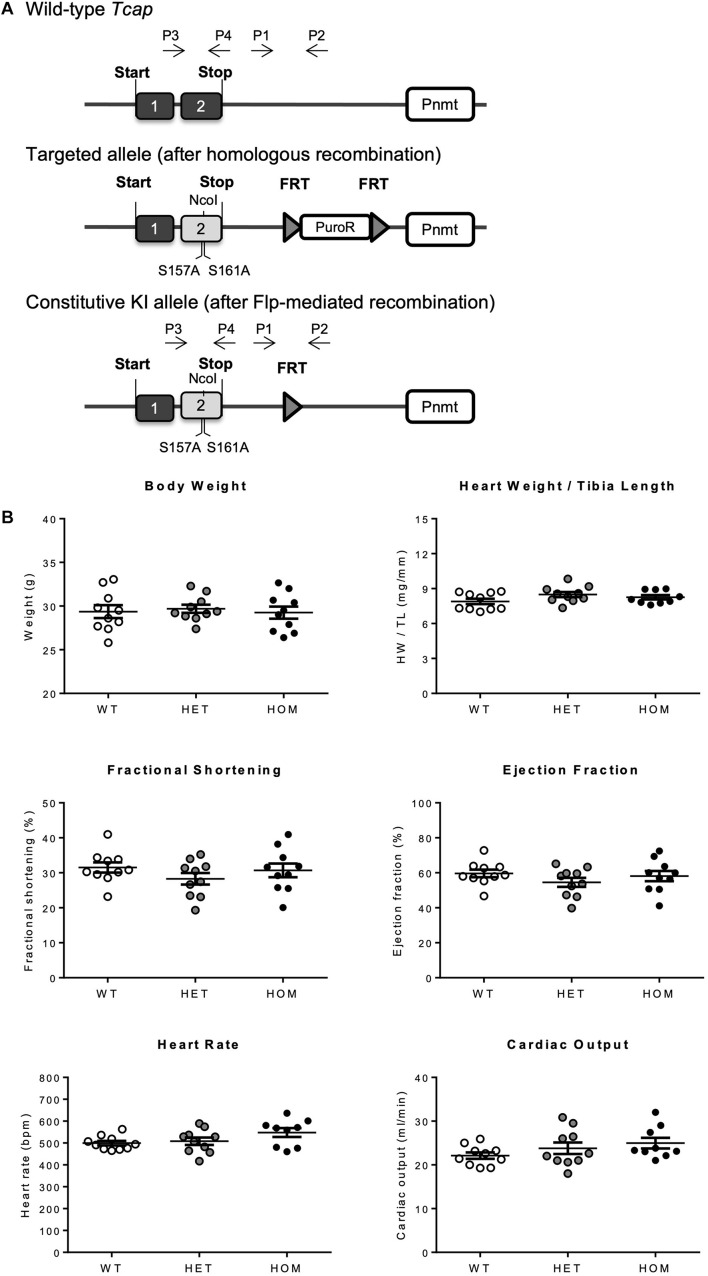
Generation and characterization of the *Tcap*^S157/161A^ KI mouse. **(A)** Schematic representation of recombination events, generating the *Tcap*^S157/161A^ KI mouse. Targeted KI allele containing puromycin resistance selection cassette (PuroR) was generated after homologous recombination, and constitutive KI allele was generated after *Flp*-mediated recombination to remove PuroR. P1–4 indicates primer binding regions, 1 and 2 indicate exons 1 and 2 of *Tcap* gene. **(B)** Baseline phenotyping of male *Tcap*^S157/161A^ KI mice. Gravimetric data from 16 week male mice including body weight and heart weight normalized to tibia length (*n* = 9–10). Echocardiographic data from 16 week male mice showing fractional shortening, ejection fraction, heart rate, and cardiac output (*n* = 9–10). Data expressed as mean ± SEM, one-way ANOVA with Tukey’s *post hoc* test.

To assess the physiological significance of telethonin phosphorylation *in vivo*, we generated a KI mouse model in which the *Tcap* gene was targeted with two single nucleotide substitutions to replace TCT codons for the phospho-acceptor S157 and S161 residues with GCT codons for non-phosphorylatable alanine (Figure 1A illustrates the gene-targeting strategy). Mice heterozygous (HET) or homozygous (HOM) for the mutated *Tcap* allele (*Tcap*^S157/161A^) were born in a Mendelian ratio, with no evidence of gestational mortality or impaired pre- or post-natal growth. Gravimetric and echocardiographic assessment of body weight, heart weight, cardiac function, and morphology at 16 weeks of age revealed no significant differences between HET and HOM mice and their WT littermates (Figure 1B; Supplementary Table 2). Similar observations were made in both male and female mice (female data not shown). These observations indicate that telethonin phosphorylation is not a critical determinant of viability and cardiac structure and function in mice, under basal conditions.

### Impact of Phosphorylation Site Ablation on Cardiac Telethonin Expression Level and Localization

**FIGURE 2:**
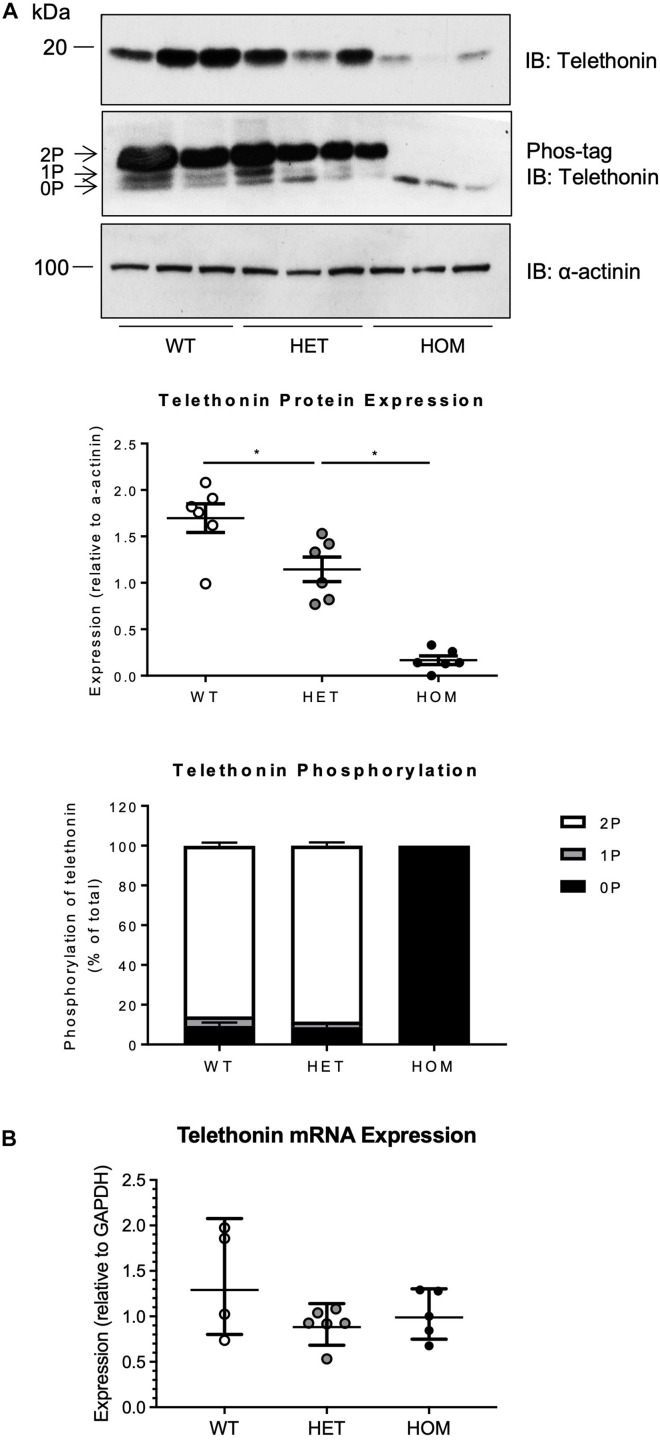
Expression and phosphorylation of telethonin in *Tcap*^S157/161A^ KI mice. **(A)** Cardiac homogenates from 16 week male mice subjected to SDS-PAGE (upper panel) and Phos-tag SDS-PAGE (middle panel) followed by immunoblotting. 2P, 1P, and 0P indicate bis-, mono-, and non-phosphorylated telethonin moieties. Densitometric analysis of telethonin protein expression normalized to α-actinin and densitometric analysis of telethonin phosphorylation normalized to the sum of the three phospho-moieties (*n* = 6). **(B)** Real-time qPCR analysis of telethonin (*Tcap*) mRNA expression in cardiac tissue from 16 week old male mice (*n* = 4–6) using the 2^–ΔΔ*CT*^ method (*Gapdh* used as a reference gene). Protein data expressed as mean ± SEM, mRNA data expressed as geometric mean ± geometric SD, *p ≤ 0.05; one-way ANOVA with Tukey’s *post hoc* test.

Telethonin expression level and phosphorylation were assessed in cardiac ventricular homogenates from WT, HET, and HOM mice at 16 weeks of age, by SDS-PAGE or Phos-tag SDS-PAGE followed by immunoblotting (Figure 2A). In WT and HET mice, cardiac telethonin existed predominantly in bis-phosphorylated (2P) form, with mono-phosphorylated (1P) and non-phosphorylated (0P) species present at much lower abundance (Figure 2A); this mirrors our earlier findings in hearts from adult WT mice and rats (Candasamy et al., 2014). In contrast, cardiac telethonin was present exclusively as the 0P species in HOM mice, as expected (Figure 2A). Interestingly, the cardiac abundance of total telethonin protein (comprising all phosphoprotein species present) was partially reduced in HET mice (67.5% expression relative to WT) and dramatically reduced in HOM mice (9.8% expression relative to WT littermates) (Figure 2A). Analysis of telethonin mRNA expression by qPCR revealed no significant difference in transcript abundance between cardiac samples from WT, HET, and HOM mice (Figure 2B). Again, similar observations were made in both male and female mice (Supplementary Figure 3). These findings indicate that ablating the telethonin phosphorylation sites at S157 and S161 has a marked impact on cardiac telethonin protein expression level and that this occurs in the absence of a change in telethonin mRNA abundance, suggesting a post-translational mechanism.

**FIGURE 3:**
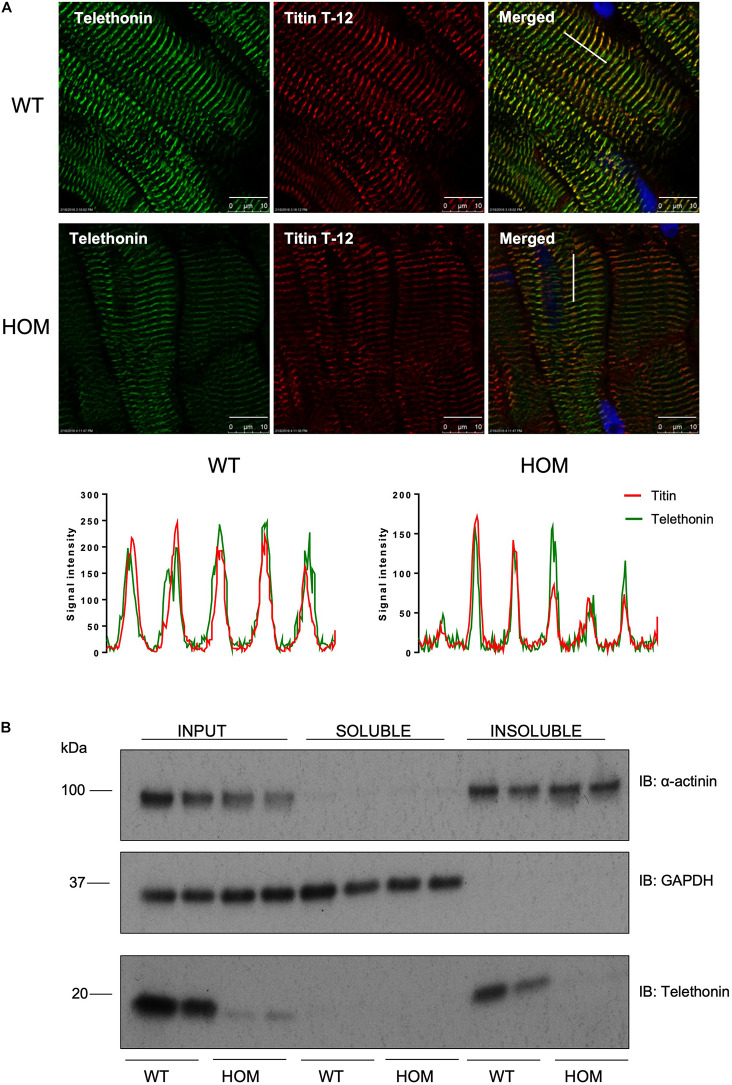
Localization of telethonin in *Tcap*^S157/161A^ KI mice. **(A)** Immunohistochemistry of cardiac tissue sections from 16 week male mice. Ten micrometers frozen cardiac tissue sections were immunostained with anti-telethonin/Alexa Fluor 488 (green) and anti-Titin N-terminal (Titin T-12)/Cy3 (red) antibodies (*n* = 6). Scale bars are 10 μm. Line charts indicate signal intensity traces of telethonin (green) and Titin T-12 (red), across the region shown by a white line shown on the merged images. **(B)** Distribution of telethonin between soluble and insoluble fractions of mouse heart homogenate in 16 week male WT and HOM mice. Samples were subjected to SDS-PAGE followed by immunoblotting with anti-α-actinin antibodies, as a marker of the insoluble myofilament fraction (upper panel), anti-GAPDH antibodies as a marker of the soluble fraction (middle panel) and anti-telethonin antibodies (lower panel).

We explored the potential impact of phosphorylation-site ablation on the subcellular distribution of telethonin protein by fluorescence confocal microscopy imaging and biochemical fractionation of cardiac samples from WT and HOM mice. Immunolabelling and confocal microscopy revealed that in ventricular myocardium of WT mice, telethonin was localized at the sarcomeric Z-disc, where it co-localized with the titin N-terminus, as expected (Figure 3A). Although telethonin signal intensity was reduced in sections from HOM hearts, reflecting the marked reduction in protein abundance (Figure 2A), Z-disc localization was not disrupted (Figure 3A). The specificity of immunolabelling with the anti-telethonin antibody was confirmed by negative control experiments with concentration-matched non-immune rabbit IgG (Supplementary Figure 4). Fractionation of myocardial samples revealed that in WT hearts telethonin was present exclusively in the Triton-insoluble fraction, where another Z-disc protein α-actinin was also exclusively localized (Figure 3B). The distribution of telethonin in HOM hearts was similar, although once again the reduced abundance of the protein in these samples was readily apparent (Figure 3B). These data show that the subcellular distribution of telethonin is not influenced by ablation of its phosphorylation sites at S157 and S161.

### Mechanism of Reduced Cardiac Telethonin Protein Expression Level in HOM Mice

**FIGURE 4:**
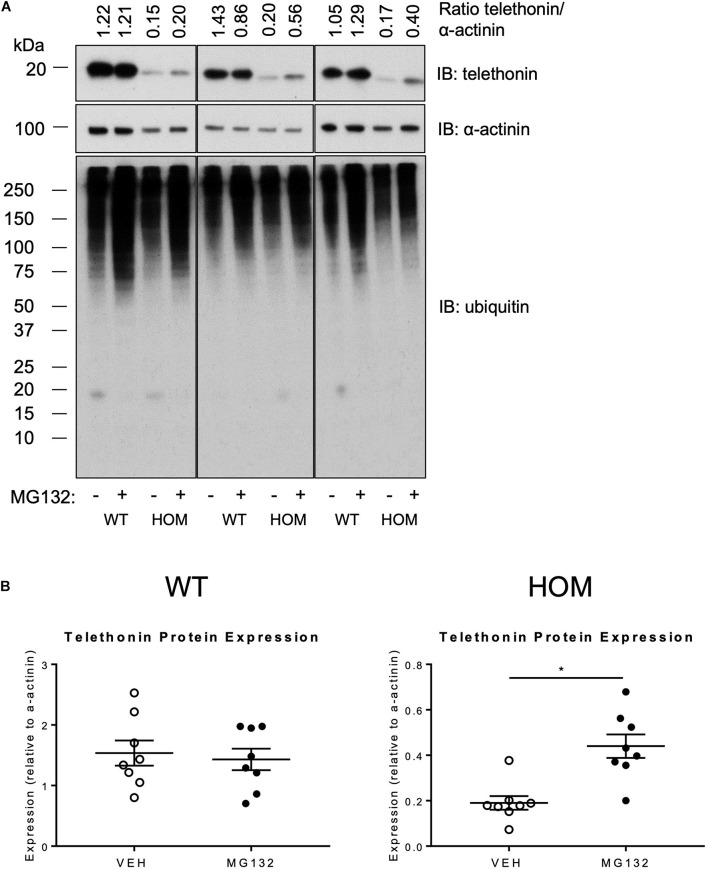
Effect of proteasome inhibition on the expression of telethonin in *Tcap*^S157/161A^ KI mice. **(A)** Immunoblot analysis of cardiomyocytes isolated from 9 to 11 week male mice treated with the vehicle DMSO (–) or 10 μm MG132 (+); representative immunoblots from experimental replicates are presented in the columns. **(B)** Densitometric analysis of telethonin expression normalized to α-actinin (*n* = 8). Data expressed as mean ± SEM, *p ≤ 0.05; unpaired *T*-test.

A potential post-translational mechanism for the reduced telethonin expression level in the hearts of HOM mice is a greater susceptibility of non-phosphorylated telethonin protein to degradation by intracellular pathways. Previous studies indicate that telethonin protein is subject to degradation by the UPS in multiple cell models (Heng et al., 2008; Polge et al., 2018; Jiang et al., 2021). To explore the potential role of the UPS system in regulating telethonin protein expression level in the presence and absence of phosphorylation at S157/S161, we determined the effects of the UPS inhibitor MG132 (Rock et al., 1994) in ventricular myocytes that were isolated from the hearts of WT and HOM mice and maintained in short-term culture. As shown in Figure 4A, MG132 treatment increased the abundance of ubiquitylated proteins, indicating effective inhibition of the proteasomal degradation of such proteins. Importantly, MG132 treatment significantly increased the expression level of S157/161A telethonin protein in myocytes from HOM mice, but not the predominantly bis-phosphorylated endogenous protein in myocytes from WT mice (Figure 4B). These findings indicate that in cardiac myocytes, non-phosphorylated telethonin protein is prone to UPS-mediated clearance at baseline, and by corollary that phosphorylation at S157/S161 may shield telethonin protein from degradation through the UPS.

### Impact of Phosphorylation-Site Ablation on the Cardiac Response to Sustained Adrenergic Stress

**FIGURE 5:**
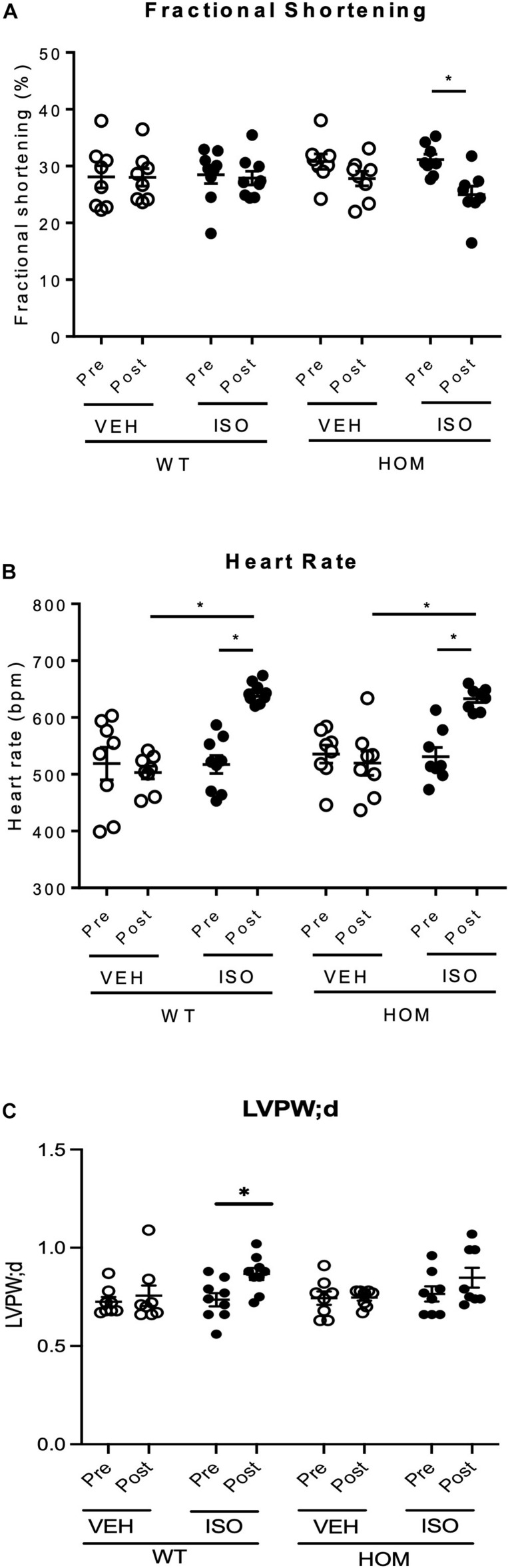
Cardiac response to sustained β-adrenergic stimulation in male *Tcap*^S157/161A^ KI mice. Male mice (8–10 weeks) were implanted with osmotic mini-pumps containing either 0.9% NaCl (VEH) or isoprenaline (ISO) at a delivery rate of 30 mg/kg/day for 14 days. Echocardiographic data shows **(A)** fractional shortening, **(B)** heart rate, and **(C)** LV posterior wall thickness in diastole (LVPW;d) in mice prior to osmotic pump implantation (Pre) and 14 days later (Post) (*n* = 8–9). Data expressed as mean ± SEM, ^∗^*p* ≤ 0.05; two-way ANOVA with Tukey’s *post hoc* test (genotype and treatment group) or Sidak’s *post hoc* test (treatment response).

To explore the potential physiological importance of telethonin phosphorylation under conditions of a sustained increase in hemodynamic load, we explored the responses of WT and HOM mice to a 2-week infusion of the β-adrenergic agonist isoprenaline (ISO), delivered through a subcutaneously implanted osmotic mini-pump (control mice received vehicle). Echocardiographic analysis was performed 1 day prior to mini-pump implantation (Pre) and repeated 2 weeks later (Post). As shown in Figure 5, left ventricular (LV) fractional shortening and ejection fraction were unaltered between the Pre and Post time points in WT and HOM mice receiving vehicle and in WT mice receiving ISO. In contrast, there was a significant decline in both fractional shortening (Figure 5A) and ejection fraction (Supplementary Table 3) in HOM mice receiving ISO. Importantly, there was a comparable chronotropic response to ISO infusion in WT and HOM mice, which displayed significant elevations in heart rate of similar magnitude (Figure 5B). As a result, a significant increase in cardiac output was observed in WT mice in response to ISO treatment, whilst cardiac output was not augmented in the HOM group (Supplementary Table 3). An increase in LV posterior wall thickness was observed in WT mice receiving ISO, however, this was not significant in HOM mice (Figure 5C). Other echocardiographic parameters did not differ significantly between groups (Supplementary Table 3). These findings suggest that ablation of telethonin phosphorylation sites impairs LV systolic function following sustained β-adrenergic stress.

**FIGURE 6:**
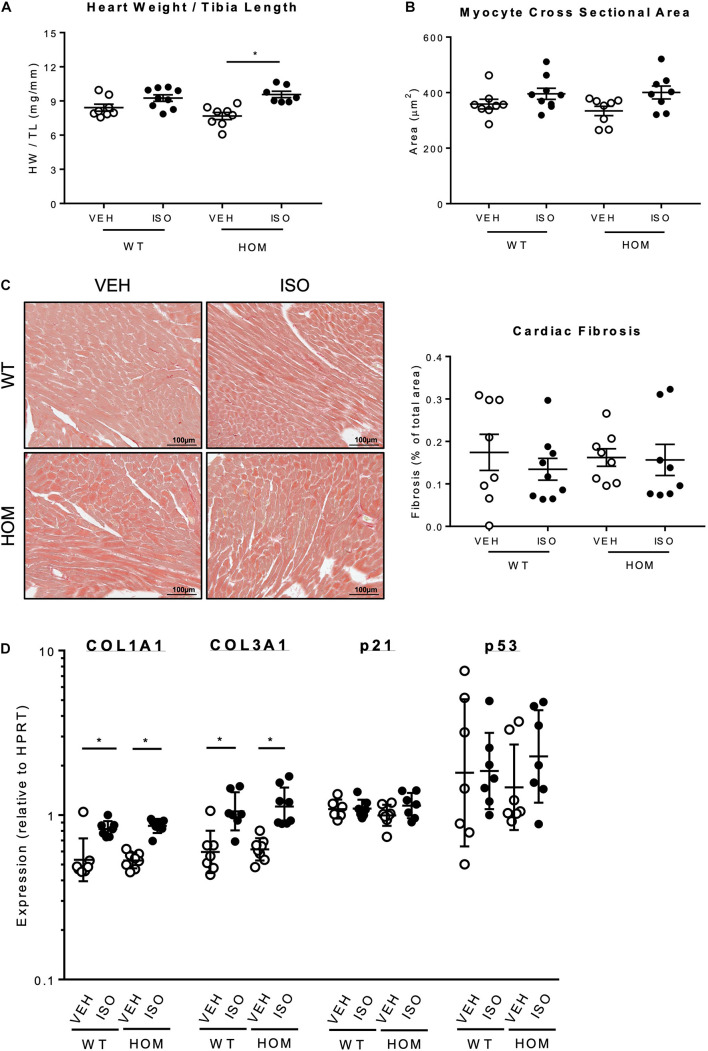
Effects of sustained β-adrenergic stimulation on cardiac remodeling in male *Tcap*^S157/161A^ KI mice. **(A)** Heart weight normalized to tibia length (*n* = 7–9). **(B)** Analysis of cardiomyocyte cross-sectional area (CSA). Five micrometers formaldehyde-fixed paraffin-embedded mouse heart sections were stained with FITC-labeled wheat germ agglutinin (WGA) to identify the myocyte cell borders and myocyte CSA was measured by using NIH ImageJ software. (CSA was measured in ≥220 myocytes per group, *n* = 8–9.) **(C)** Representative images of Picro Sirius Red staining of paraffin-embedded mouse heart sections (left panel). Quantification of cardiac fibrosis determined by Picro Sirius Red staining (right panel) (*n* = 8–9). Scale bars are 100 μm. **(D)** Real-time qPCR analysis of mRNAs associated with fibrosis (*Col1a1*, *Col3a1*) and apoptosis (*p21*, *p53*) in cardiac tissue using the 2^–ΔΔ*CT*^ method (*Hprt* used as a reference gene) (*n* = 7–8). Heart weight to tibia length ratio, myocyte cross sectional area, and fibrosis quantification expressed as mean ± SEM, mRNA data expressed as geometric mean ± geometric SD, *p ≤ 0.05; two-way ANOVA with Tukey’s *post hoc* test.

Post-mortem analysis of hearts after the 2-week infusion period revealed a significantly greater heart weight/tibia length ratio in HOM mice that received ISO relative to HOM mice that received vehicle (Figure 6A), indicating ISO-induced cardiac hypertrophy. In contrast, there was no significant difference in heart weight/tibia length ratio between WT mice that received vehicle and those that received ISO (Figure 6A), suggesting that the hypertrophic response to sustained β-adrenergic stress was exaggerated in HOM mice. A similar trend was observed with respect to ISO-induced increases in myocyte cross sectional area, which tended to be greater in HOM mice than in WT mice (Figure 6B), although the inter-group differences were not statistically significant. To explore whether there was any indication of pathological cardiac remodeling in response to ISO infusion in mice of either genotype, we performed histological analysis of ventricular sections using Sirius Red to label collagen. This revealed minimal labeling in WT and HOM mice infused with either vehicle or ISO, indicating the absence of significant cardiac fibrosis (a hallmark of pathological remodeling) in all four groups (Figure 6C). Nevertheless, qPCR analysis revealed small but significant ISO-induced increases in the expression of the collagen genes *Col1a1* and *Col3a1* in both WT and HOM mice, with no significant difference between the genotypes in the ISO response (Figure 6D). There was no induction of the pro-apoptotic gene *p53*, or the p53 target gene *p21*, in response to ISO infusion in either WT or HOM mice (Figure 6D). Taken together, these data indicate that in response to a sustained β-adrenergic stress protocol that induces a mild phenotype in WT mice, HOM mice exhibit a more pronounced hypertrophic response, but without evidence of pathological remodeling.

**FIGURE 7:**
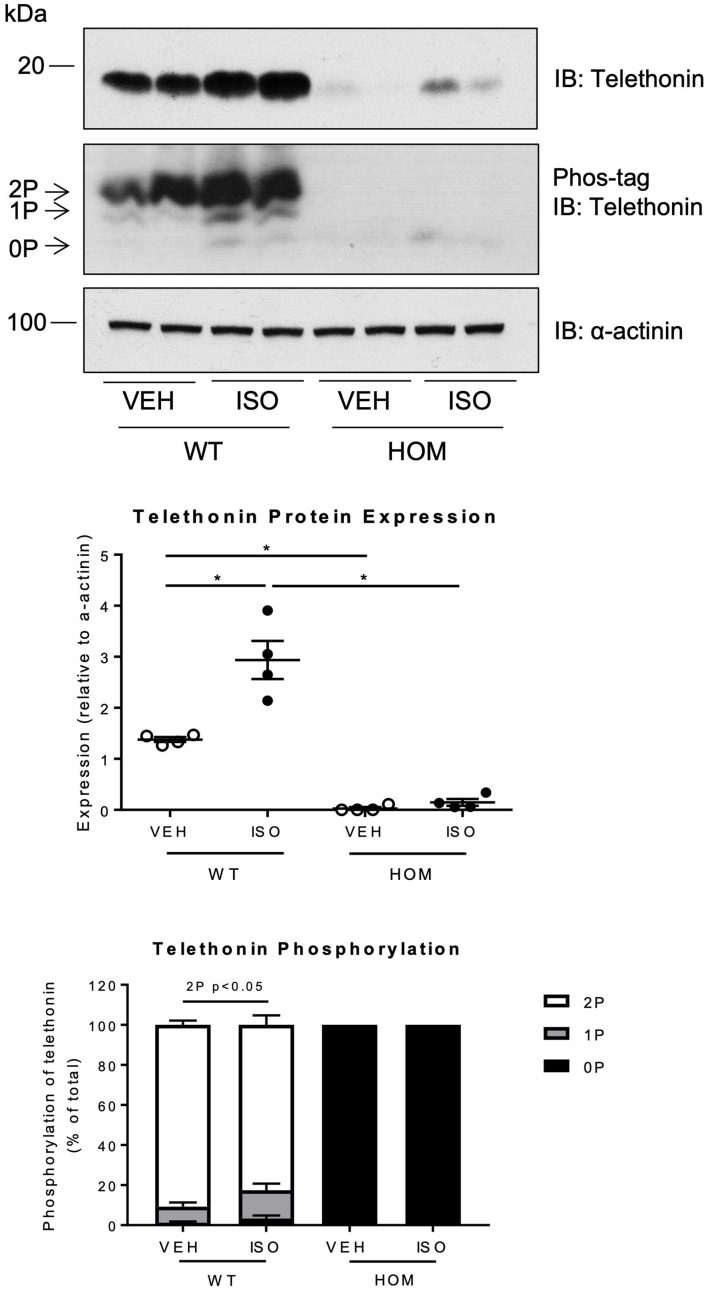
Effects of sustained β-adrenergic stimulation on telethonin expression and phosphorylation in male *Tcap*^S157/161A^ KI mice. Immunoblot analysis of heart homogenates subjected to SDS-PAGE or Phos-tag SDS-PAGE. 2P, 1P, and 0P indicate bis-, mono-, and non-phosphorylated telethonin moieties. Densitometric analysis of immunoblots (*n* = 4) (middle panel). Densitometric analysis of telethonin phosphorylation normalized to the sum of the three phospho-moieties (*n* = 4) (lower panel). Data expressed as mean ± SEM, *p ≤ 0.05; two-way ANOVA with Tukey’s *post hoc* test.

We also determined the expression level and phosphorylation status of telethonin in ventricular myocardium from WT and HOM mice that received a 2-week infusion of vehicle or ISO. Interestingly, ISO infusion led to a large increase in telethonin protein expression levels in the hearts of WT mice but *not* those of HOM mice (Figure 7). In contrast, the expression levels of two other sarcomeric proteins, the inhibitory subunit of cardiac troponin (cTnI) and cardiac myosin-binding protein C (cMyBP-C) were unchanged by ISO infusion in both WT and HOM mice (Supplementary Figure 7B). Cardiac telethonin was expressed predominantly in bis-phosphorylated form in WT mice and exclusively in non-phosphorylated form in HOM mice, regardless of the infusion of vehicle or ISO; these findings indicate that phosphorylation at Ser157 and Ser161 is maintained during sustained β-adrenergic stimulation and that such stimulation does not induce telethonin phosphorylation at other sites. Despite the ISO-induced increase in telethonin protein expression level in ventricular myocardium from WT mice (Figure 7), there was no difference in telethonin mRNA expression between the groups (Supplementary Figure 7A). Furthermore, subcellular distribution of telethonin, as assessed by immunolabelling and myocardial fractionation, is not affected despite an increase in telethonin protein expression level in response to ISO infusion (Supplementary Figures 5A,B). These findings reveal that the cardiac response to ISO infusion in WT mice includes a marked increase in the expression level of telethonin protein, but not telethonin mRNA, and that this increase requires that Ser157 and Ser161 are available for phosphorylation.

### Impact of Phosphorylation Site Ablation on the Cardiac Response to Acute Adrenergic Stimulation

The differential cardiac responses of WT and HOM mice to sustained β-adrenergic stress led us to determine whether ablation of telethonin phosphorylation sites impacts also on cardiac responses to acute β-adrenergic stimulation. To that end, serial echocardiographic assessment of cardiac function was undertaken in WT and HOM mice at baseline, and 2–3 min after vehicle administration (VEH), then ISO administration (ISO), with both VEH and ISO administered by intraperitoneal injection in an identical volume. As illustrated in Supplementary Figure 6, ISO injection induced comparable increases in fractional shortening, ejection fraction, and heart rate in both WT and HOM mice. These findings indicate that ablation of telethonin phosphorylation sites does not impact on the positive inotropic and positive chronotropic responses of the mouse heart to acute β-adrenergic stimulation *in vivo*.

## Discussion

This study presents the following novel observations: (1) ablation of telethonin phosphorylation sites S157 and S161 *in vivo* results in a gene-dosage-dependent decrease in cardiac telethonin protein expression level, in the absence of any change in telethonin mRNA expression; (2) the reduction in telethonin protein expression level appears to arise from UPS-mediated clearance of non-phosphorylated telethonin protein; (3) telethonin protein expression level is increased in WT but not HOM mice following sustained β-adrenergic stress; and (4) telethonin phosphorylation site ablation impairs LV systolic function in response to sustained β-adrenergic stimulation, without evidence of pathological remodeling.

To date, the functional role of cardiac telethonin and the *in vivo* physiological significance of the constitutive phosphorylation of its C-terminal S157 and S161 residues have remained unknown. To address this, we have generated and investigated a novel non-phosphorylatable (S157/161A) telethonin KI mouse model. Characterization of this mouse model found that ablation of telethonin phosphorylation did not impact the basal cardiac phenotype in mice heterozygous or homozygous for the mutated *Tcap* allele. In a previously published *Tcap* knockout mouse model, no alterations in cardiac parameters were revealed at baseline, suggesting that telethonin is not necessary for the maintenance of murine cardiac function in unstressed, physiological conditions (Knoll et al., 2011). Moreover, ablation of the phosphorylation sites S157 and S161 did not modify the subcellular distribution of telethonin, as the mutated protein remained co-localized with the titin N-terminus at the sarcomeric Z-disc and was exclusively detected in the insoluble sarcomeric fraction. Interestingly, however, a gene dosage-dependent decrease in telethonin protein expression level was revealed in HET and HOM mice that was independent of any changes in *Tcap* expression at mRNA level. We postulate that the observed discrepancy between telethonin protein and mRNA expression is a result of altered post-translational regulation and that the absence of telethonin phosphorylation at S157 and S161 influences protein stability.

Previous studies have identified telethonin as a putative substrate of the 26S proteasome and three E3 ubiquitin-ligase enzymes, MDM2 and MURF1 and MURF2, have been shown to interact with and down-regulate telethonin (Witt et al., 2005; Tian et al., 2006; Heng et al., 2008; Polge et al., 2018). Proteasome inhibition has also been shown to inhibit MDM2-mediated degradation of telethonin in H1299 cells (Tian et al., 2006). Furthermore, a recent study has identified telethonin as a target of the proteasome in a titin missense variant mouse model of cardiomyopathy, where degradation of unbound, cytoplasmic telethonin by the proteasome results in proteasomal overload (Jiang et al., 2021). In line with these observations, our study has identified UPS as an important post-translational mechanism responsible for the reduced telethonin expression level in the HOM mice; the proteasome inhibitor MG132 was found to increase significantly the expression level of S157/161A telethonin protein in myocytes from HOM mice, but not the expression level of phosphorylatable telethonin protein in myocytes from WT mice. A potential telethonin ubiquitylation site has been identified at K148 (Wagner et al., 2012), in proximity to the two C-terminal phosphorylation sites at S157 and S161. This raises the possibility that bis-phosphorylation of the telethonin C-terminus at these sites could prevent ubiquitylation at K148, such that the non-phosphorylated protein expressed in KI mice becomes more susceptible to E3-mediated ubiquitylation at K148 and thus UPS-mediated degradation.

The sustained β-adrenergic stimulation model (isoprenaline infusion) has been previously shown to elicit a hypertrophic response and increased cardiac contractility in mice following a 2-week treatment (Puhl et al., 2016). In our study, in response to isoprenaline infusion, only HOM mice presented a hypertrophic phenotype that was accompanied by systolic dysfunction, as reflected by reduced fractional shortening, ejection fraction, and cardiac output. These findings indicate that the absence of telethonin phosphorylation exacerbates the cardiac remodeling response and impairs LV systolic function following sustained β-adrenergic stress. This is similar to the cardiac phenotype previously reported in *Tcap* KO mice 2–3 weeks after transverse aortic constriction (TAC) (Knoll et al., 2011), which is characterized by LV hypertrophy, increased end-diastolic and end-systolic LV diameter and reduced fractional shortening. Thus, it is likely that the cardiac phenotype displayed in our KI model following sustained β-adrenergic stimulation arises from the markedly reduced telethonin protein expression levels, rather than directly from reduced telethonin phosphorylation. Importantly, acute isoprenaline administration *via* intraperitoneal injection was found to induce comparable inotropic and chronotropic responses in WT and HOM mice, indicating that the β-adrenergic signaling pathway remains intact in the latter.

In an effort to identify underlying mechanisms that contribute to more pronounced hypertrophy and systolic dysfunction in our KI model following sustained β-adrenergic stimulation, we investigated potential telethonin-regulated pathways highlighted by previous work. However, we did not observe any significant differences in the induction of pro-apoptotic genes (p53, p21) in response to chronic ISO treatment (Knoll et al., 2011), or in calcium transient dynamics in isolated myocytes from WT and KI mice (data not shown) (Ibrahim et al., 2013; Candasamy et al., 2014). It is possible that proteasomal overload due to increased degradation of mutant telethonin protein in our KI model may contribute to the observed cardiac phenotype (Jiang et al., 2021), and further studies are warranted to delineate the principal mechanisms.

It is notable that sustained β-adrenergic stimulation was accompanied by a substantial increase in cardiac telethonin protein expression levels exclusively in WT mice, in which the protein remained predominantly bis-phosphorylated. Telethonin appears to be unique in this response, as the expression levels of myofilament proteins cTnI and cMyBP-C were not altered. The increase in telethonin protein expression level was found to be independent of changes in *Tcap* mRNA transcript expression, again suggesting a role for altered post-translational regulation. In the absence of differential mRNA expression, increased expression of cytoskeletal proteins has been previously reported in cardiac hypertrophy (Liu et al., 2016), mediated through enhanced mRNA translation *via* the protein complex mTORC1. The augmented telethonin protein expression level also appears to be phosphorylation-dependent; it is possible that in HOM mice the translation of *Tcap* mRNA is increased but the newly synthesized non-phosphorylatable telethonin is rapidly targeted for UPS-mediated degradation, as discussed above. This may be reflected by the observation that the telethonin mRNA is one of the most abundant mRNAs in striated muscle (Valle et al., 1997) and suggests dynamic translational regulation of this mRNA pool.

Telethonin mutations are associated with hypertrophic and dilated cardiomyopathies, and the muscular dystrophy LGMD2G. In many clinical reports of LGMD2G, the mutations in telethonin result in a premature stop codon and predicted truncation of the C-terminus. However, an E82X mutation was reported in a recent study, in which a 10 kDa telethonin protein was detected and localized at the sarcomeric Z-disc (Barresi et al., 2015). This study provided the first evidence of an LGMD2G-associated telethonin mutation resulting in a truncated N-terminal protein lacking the C-terminal phosphorylation sites. Conversely, telethonin mutations associated with cardiomyopathies frequently result in point mutations, rather than premature stop codons. C-terminal telethonin mutations (i.e., R153H and R158C) have been identified in close proximity to the two phosphorylation sites (Hayashi et al., 2004; Hirtle-Lewis et al., 2013). *In vitro* modeling of the R153H mutation indicated that the mutation compromised the binding of telethonin with titin and calsarcin-1 (Hayashi et al., 2004), yet mutations in this region could also potentially impair the phosphorylation of residues S157 and S161. In the context of dilated cardiomyopathy (DCM), the point mutation at R158 could influence telethonin phosphorylation at S161, as the kinases PKD and CaMKII exhibit a preference for arginine at position −3 of the phospho-acceptor residue (Nishikawa et al., 1997; Swulius and Waxham, 2008). The R158C mutation replaces an amino acid with a basic side chain with a polar neutral residue, thus potentially impacting on the kinase binding kinetics. Investigation of the impact of disease-associated telethonin mutations on telethonin phosphorylation and thereby stability could provide novel mechanistic insight into pathogenesis. Furthermore, the role of telethonin phosphorylation during muscle remodeling under increased activity of the UPS, e.g., in muscle atrophy in critically ill patients, paralysis, or in the unloaded heart, will now be interesting to investigate.

In conclusion, the present study has assessed the functional significance of telethonin bis-phosphorylation in a murine model and has identified that telethonin protein turnover is regulated in a novel phosphorylation-dependent manner. We postulate that telethonin turnover is regulated by the UPS and that C-terminal phosphorylation may protect against UPS-mediated degradation. Cardiac telethonin protein expression level was increased in WT mice in response to sustained β-adrenergic stress, but this response was absent in HOM mice, in which telethonin phosphorylation site ablation impaired LV systolic function. Taken together, our new data suggest that the phosphorylation state of telethonin strongly influences the protein’s stability, and that regulation of telethonin protein expression level may be critical to the preservation of cardiac function during sustained hemodynamic stress.

## Data Availability Statement

The original contributions presented in the study are included in the article/Supplementary Material, further inquiries can be directed to the corresponding author.

## Ethics Statement

The animal study was reviewed and approved by King’s College London local ethics review board.

## Author Contributions

HL and SE made a significant contribution to the conception and design of the project, the acquisition, analysis, interpretation of data, and jointly drafted the manuscript. MG made a significant contribution to the design of the project and reviewed and revised the manuscript critically for important intellectual content. MA was the last author and made a significant contribution to the conception and design of the project and reviewed and revised the manuscript critically for important intellectual content. All authors contributed to the article and approved the submitted version.

## Conflict of Interest

The authors declare that the research was conducted in the absence of any commercial or financial relationships that could be construed as a potential conflict of interest.

## Publisher’s Note

All claims expressed in this article are solely those of the authors and do not necessarily represent those of their affiliated organizations, or those of the publisher, the editors and the reviewers. Any product that may be evaluated in this article, or claim that may be made by its manufacturer, is not guaranteed or endorsed by the publisher.

## Footnotes

1
https://pga.mgh.harvard.edu/primerbank/
