# Supplementary material for: Phosphorylation at Serines 157 and 161 Is Necessary for Preserving Cardiac Expression Level and Functions of Sarcomeric Z-Disc Protein Telethonin
Source: Front Physiol. 2021 Sep 8;12:732020. doi: 10.3389/fphys.2021.732020 (PMC8455888; doi:10.3389/fphys.2021.732020)
Supplement: Supplementary file 1 [file Data_Sheet_1.pdf]

Supplementary Material

1 Supplementary Methods

Generation of *Tcap*$^{S157/161A}$ KI mice

The amino acid substitutions S157A and S161A were introduced into the mouse *Tcap* gene by PCR mutagenesis and the mutant *Tcap* sequence was inserted into a targeting plasmid upstream of a puromycin resistance cassette, flanked by FRT sites (Figure 1A). Mouse embryonic stem cells (ES) isolated from C57BL/6N were electroporated with the targeting vector; puromycin-resistant ES clones were isolated and screened for homologous recombination by Southern blot analysis. Puromysin-resistant ES clones were injected into 3.5 dpc blastocysts before transfer to pseudo-pregnant BALB/c females. Highly chimeric offspring (G0) were bred with C57BL/6N females, homozygous for the *Flp*-deleter, generating black C57BL/6N offspring (G1) with the puromycin resistance cassette excised.

Genotyping of *Tcap*$^{S157/161A}$ KI mice

PCR primers were designed to amplify genomic DNA spanning the inserted FRT site (P1: 5' GGACACGATTGTTGACTGAGG 3', and P2: 5' GAGATAAGGAAGGTGAGCCG 3') and spanning the two point mutations (P3: 5' TCTTCACGCCCACCAAGG 3'and P4: 5' AGGACACCTAGGCATCCTGG 3'). PCR with primer pair P1-P2 yields a 386 bp amplicon (KI allele) and 311 bp amplicon (WT allele). Amplicons (387 bp) from primer pair P3-P4 were digested with NcoI at 37°C for one hour to yield 199 bp and 188 bp products (KI allele). PCR products were separated on 2% agarose-TAE gels (Supplementary Figure 1A and B). During the generation of the mouse, a puromycin-resistance cassette, flanked by two FRT sites, was inserted downstream of the mutated *Tcap* gene (Figure 1A). This insertion is proximal to the phenylethanolamine N-methyltransferase gene (*Pnmt*). To ensure that this insertion did not have any unintended effects on the expression of this proximal gene, qPCR analysis of *Pnmt* mRNA was performed. Our results show that *Pnmt* mRNA expression (relative to *Hprt*) was not altered in the *Tcap* S157/161A KI mice, in either males or females (Supplementary Figure 2A and B).

Tissue processing for SDS-PAGE and immunoblotting

Animals were euthanised by cervical dislocation, following isoflurane-induced anaesthesia (3-4% isoflurane with 96-97% O2 at a flow rate of 1 L/min) and tissues were snap-frozen in liquid nitrogen. Frozen ventricular tissues were pulverised and then homogenised in lysis buffer (50 mmol/L Tris HCl, (pH 7.4), 137 mmol/L NaCl, 10% glycerol, 20 mmol/L NaF, 2 mmol/L NaV, 1% Triton X-100, 1% v/v Phosphatase Inhibitor Cocktail 3 (Sigma) and 0.1% EDTA-free protease inhibitor tablet (Roche)). Samples were denatured by heating to 95°C for 5 minutes in sample buffer (1.9 mol/L urea, 67.3 mmol/L Tris-HCl (pH 6.8), 2.7% SDS, 1.2% Igepal, 2.23% β-mercaptoethanol, 2% glycerol and 3% bromophenol blue). For subcellular fractionation, heart homogenates were centrifuged at 18000 g for 30 minutes at 4°C, as described previously.[1] Supernatant was collected (soluble fraction) and denatured in sample buffer, as described above. The pellet (insoluble fraction) was solubilised in sample buffer, made to equal volumes as the soluble fraction with lysis buffer,

before denaturing at 95°C. Samples were then resolved by SDS-PAGE for subsequent immunoblotting.

RNA isolation and quantitative-PCR

Total RNA was isolated from frozen pulverised tissues with mirVana™ RNA Isolation Kit (ThermoFisher) or RNEasy RNA isolation kit (Qiagen). Total RNA was quantified using Nanodrop 2000 Spectrophotometer (ThermoFisher) and RNA purity was assessed by A_{260}/A_{280}. 250-500 ng total RNA was reverse transcribed using 20 umol/L random hexamers (ThermoFisher) to obtain cDNA using Omniscript RT Kit (Qiagen), according to the manufacturer's instructions. A reaction where reverse transcriptase was omitted was also set-up in parallel to assess for any genomic DNA contamination during subsequent qPCR amplification.

Isolation and culture of murine cardiomyocytes

Mice were anaesthetised by intraperitoneal injection of 20% sodium pentobarbitone (Animalcare) at a dose of 36 µg/kg body weight, supplemented with sodium heparin. Hearts were perfused by retrograde perfusion with modified Tyrode's solution (130 mmol/L NaCl, 1.4 mmol/L $MgCl_2$, 5 mmol/L HEPES, 0.4 mmol/L NaH_2PO_4, 5.4 mmol/L KCl, 10 mmol/L creatine, 20 mmol/L taurine, 10 mmol/L glucose, pH 7.3), supplemented with 0.75 mmol/L Ca^{2+}. The perfusate was switched to Ca^{2+}-free modified Tyrode's solution containing 0.2 mmol/L EGTA for 5 minutes. Digestion was performed by perfusion with modified Tyrode's solution containing 0.1 mmol/L Ca^{2+} and 0.7-0.8 mg/ml type II collagenase (Worthington Biochemical) for 12 minutes. Homogenates were filtered through a 200 µm nylon mesh then re-suspended in 0.5 mmol/L Ca^{2+} modified Tyrode's solution supplemented with 10 mg/ml albumin. Myocytes were then re-suspended in 1 mmol/L modified Tyrode's solution. Following isolation, cardiomyocytes were re-suspended in pre-warmed M199 culture medium (Invtirogen) supplemented with 2 mmol/L creatine, 5 mmol/L taurine, 2 mmol/L carnitine and 1% (v/v) Penicillin-Streptomycin (Invitrogen). Cells were plated on 6-well culture plates, pre-prepared by coating with 15 µg/ml laminin (Sigma L2020) and maintained at 37°C, 5% CO_2.

References

1. Weeks KL, Ranieri A, Karas A, Bernardo BC, Ashcroft AS, Molenaar C, *et al.* beta-Adrenergic Stimulation Induces Histone Deacetylase 5 (HDAC5) Nuclear Accumulation in Cardiomyocytes by B55alpha-PP2A-Mediated Dephosphorylation. *J Am Heart Assoc* 2017;**6**.

2 Supplementary Figures and Tables

2.1 Supplementary Figures

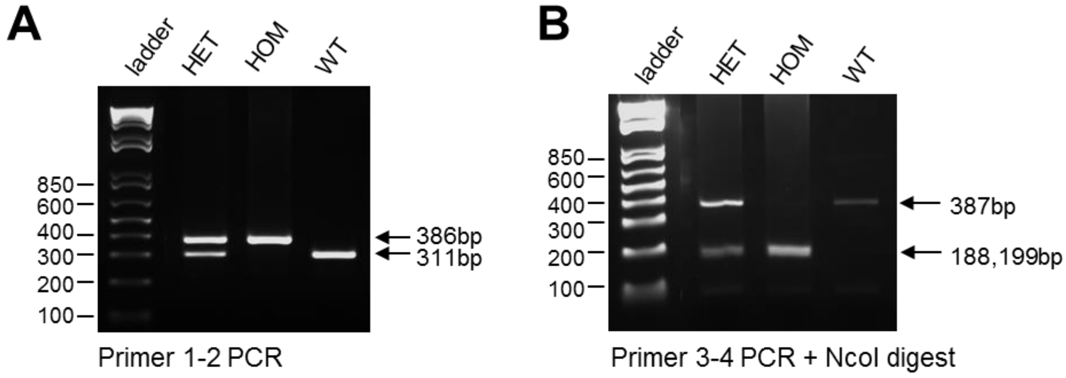

Supplementary Figure 1. Genotyping strategy for *Tcap*$^{S157/161A}$ KI mouse. **(A)** Primer P1-2 PCR yields 311 bp amplicon for WT allele and 386bp amplicon for KI allele. **(B)** Primer P3-4 PCR followed by digestion with NcoI restriction enzyme at 37°C for 1 hour yields 387 bp amplicon for WT allele (not digested) and 188/199 bp amplicons for KI allele.

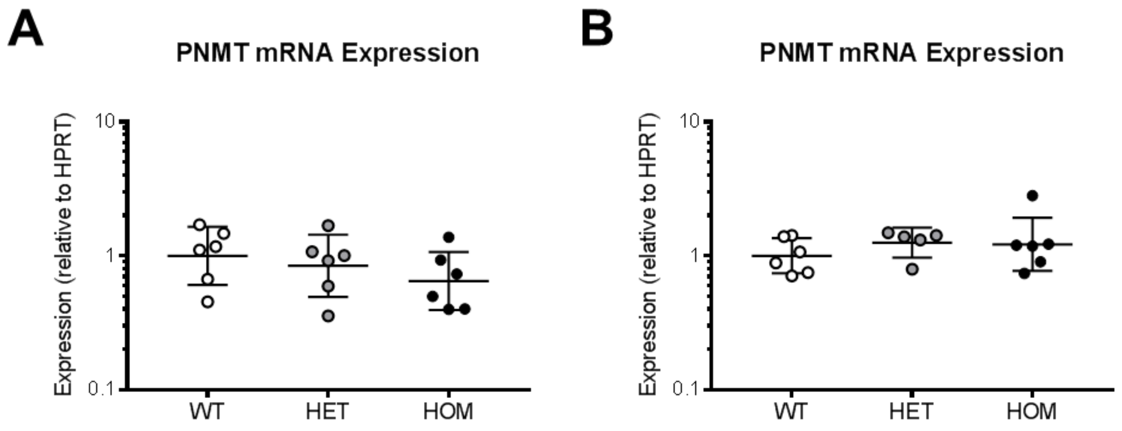

Supplementary Figure 2. Expression levels of phenylethanolamine N-methyltransferase (*Pnmt*) in *Tcap*$^{S157/161A}$ KI mouse. Real-time qPCR analysis of *Pnmt* mRNA expression in adrenal gland tissue in 16 week old **(A)** male and **(B)** female mice (n=5/6) using the $2^{-\Delta\Delta CT}$ method (*Hprt* used as a reference gene). Data expressed as geometric mean ± geometric SEM; 1-way ANOVA with Tukey's post hoc test.

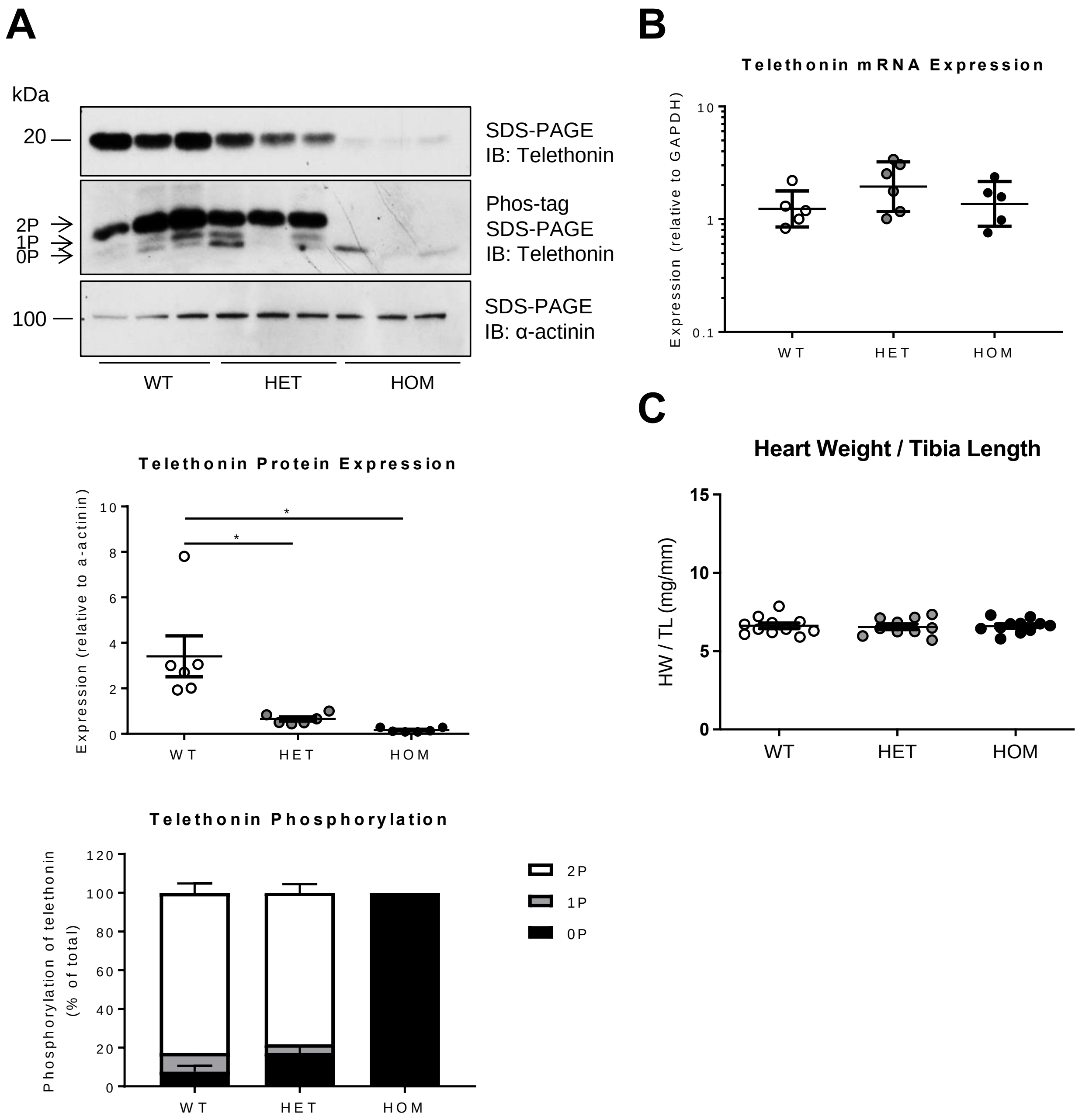

Supplementary Figure 3. Expression and phosphorylation of telethonin in *Tcap*$^{S157/161A}$ KI mouse. **(A)** Cardiac homogenates from 16 week female mice were subjected to SDS-PAGE and Phos-tag SDS-PAGE followed by immunoblotting. 2P, 1P and 0P indicate bis-, mono- and non-phosphorylated telethonin moieties. Densitometric analysis of telethonin immunoblots is shown in lower panel (n=6). Densitometric analysis of telethonin phosphorylation normalised to the sum of the three phospho-moieties (n=6) **(B)** Real-time qPCR analysis of telethonin mRNA expression in cardiac tissue from 16 week old female mice (n=5-6) using the $2^{-\Delta\Delta CT}$ method (*Gapdh* used as a reference gene). (**C**) Heart weight normalised to tibia length in 16 week old female mice (n=10-11). Protein and gravimetric data expressed as mean ± SEM, mRNA data expressed as geometric mean ± geometric SEM, * $p \leq 0.05$; 1-way ANOVA with Tukey's post hoc test.

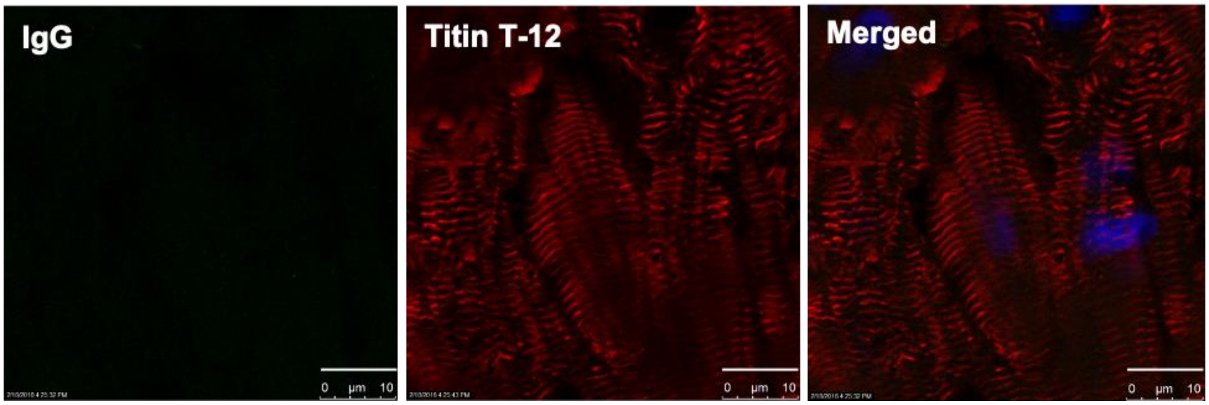

Supplementary Figure 4. Negative control for telethonin immunohistochemistry. Frozen cardiac tissue sections from wild-type mice were incubated with anti-N-terminal Titin (T-12) antibodies (red) and rabbit IgG (green) at a concentration matched to the rabbit monoclonal telethonin antibody. Scale bars are 10 µm.

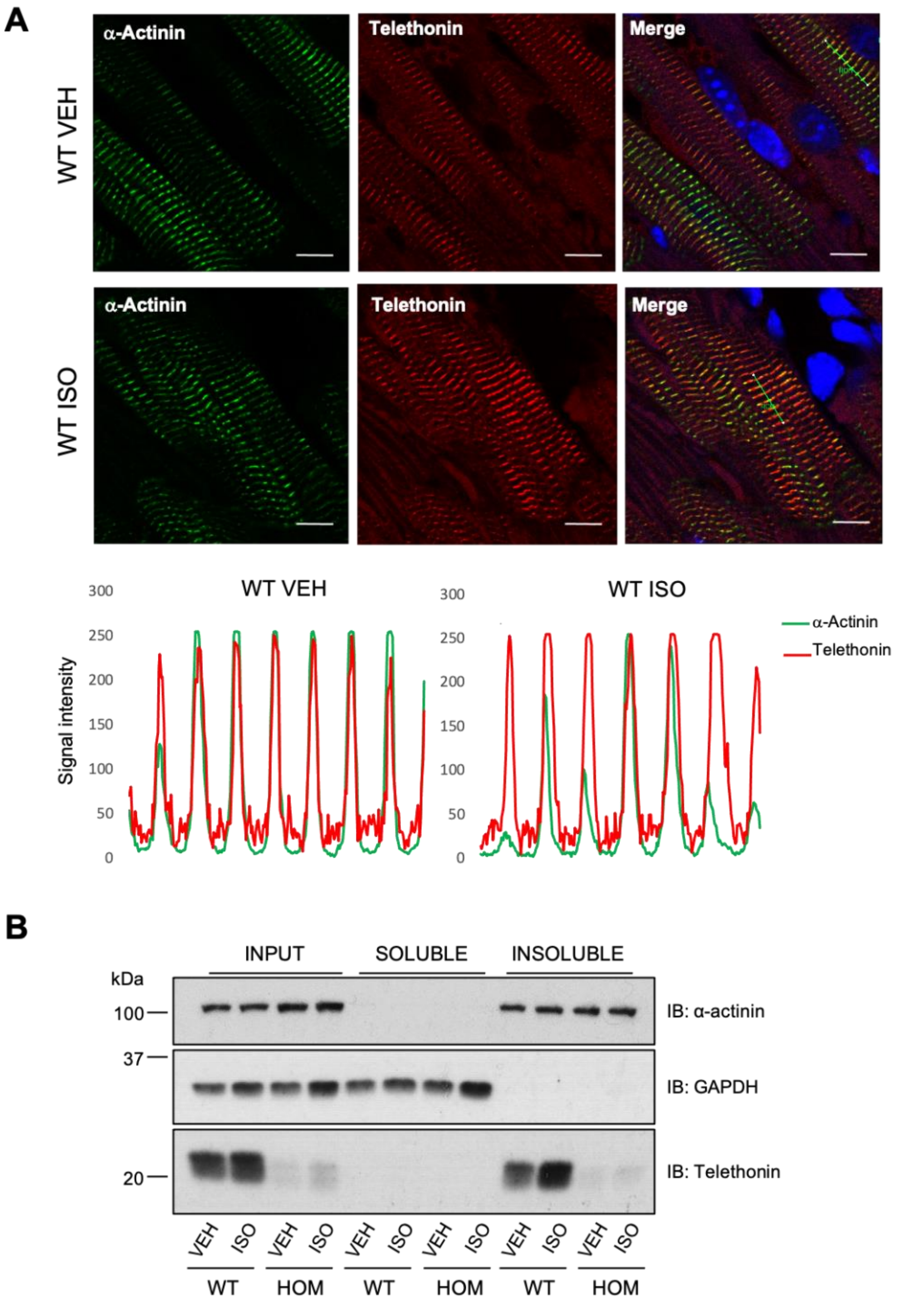

Supplementary Figure 5: Effects of sustained β-adrenergic stimulation on telethonin localisation in male *Tcap*$^{S157/161A}$ KI mice. **(A)** 10 µm paraffin cardiac tissue sections were immunostained with anti-alpha-Actinin (green) and anti-Telethonin (red) antibodies (n=3). Scale bars are 7.5 µm.
(B) Distribution of telethonin between soluble and insoluble fractions of mouse heart homogenate in 10-12 week male WT and HOM mice, treated with VEH or ISO. Samples were subjected to SDS-PAGE followed by immunoblotting with anti-α-actinin antibodies, as a marker of the insoluble myofilament fraction (upper panel), anti-GAPDH antibodies as a soluble fraction marker (middle panel) and anti-telethonin antibodies (lower panel).

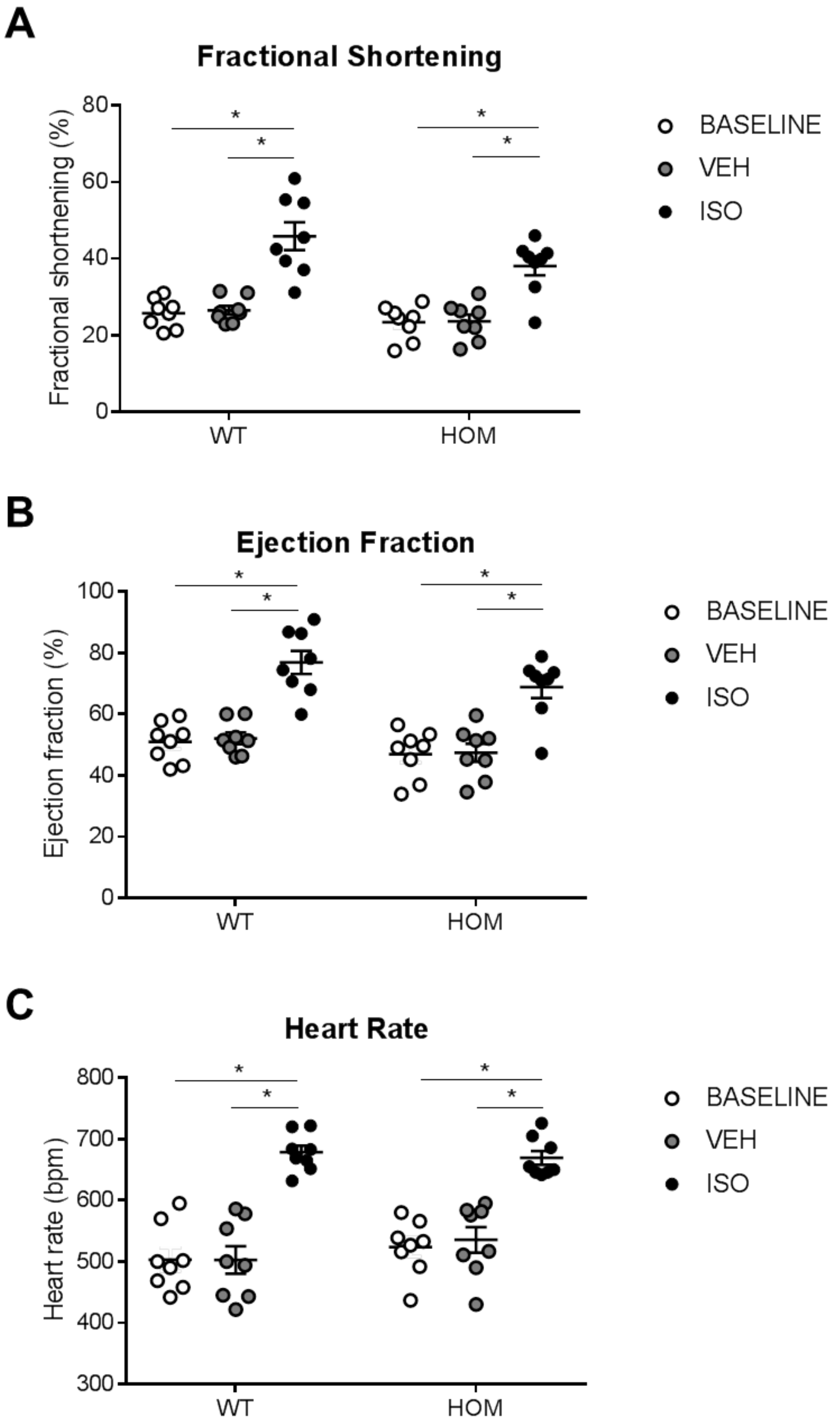

Supplementary Figure 6: Cardiac response to acute β-adrenergic stimulation in male *Tcap*$^{S157/161A}$ KI mice. Echocardiographic data shows **(A)** fractional shortening, **(B)** ejection fraction and **(C)** heart rate in mice at baseline and following intraperitoneal injections of 0.9% NaCl and isoprenaline hydrochloride (0.1 mg/kg). Data expressed as mean ± SEM, * $p \leq 0.05$; 2-way ANOVA with Tukey's post hoc test (treatment response) or Sidak's post hoc test (genotype response).

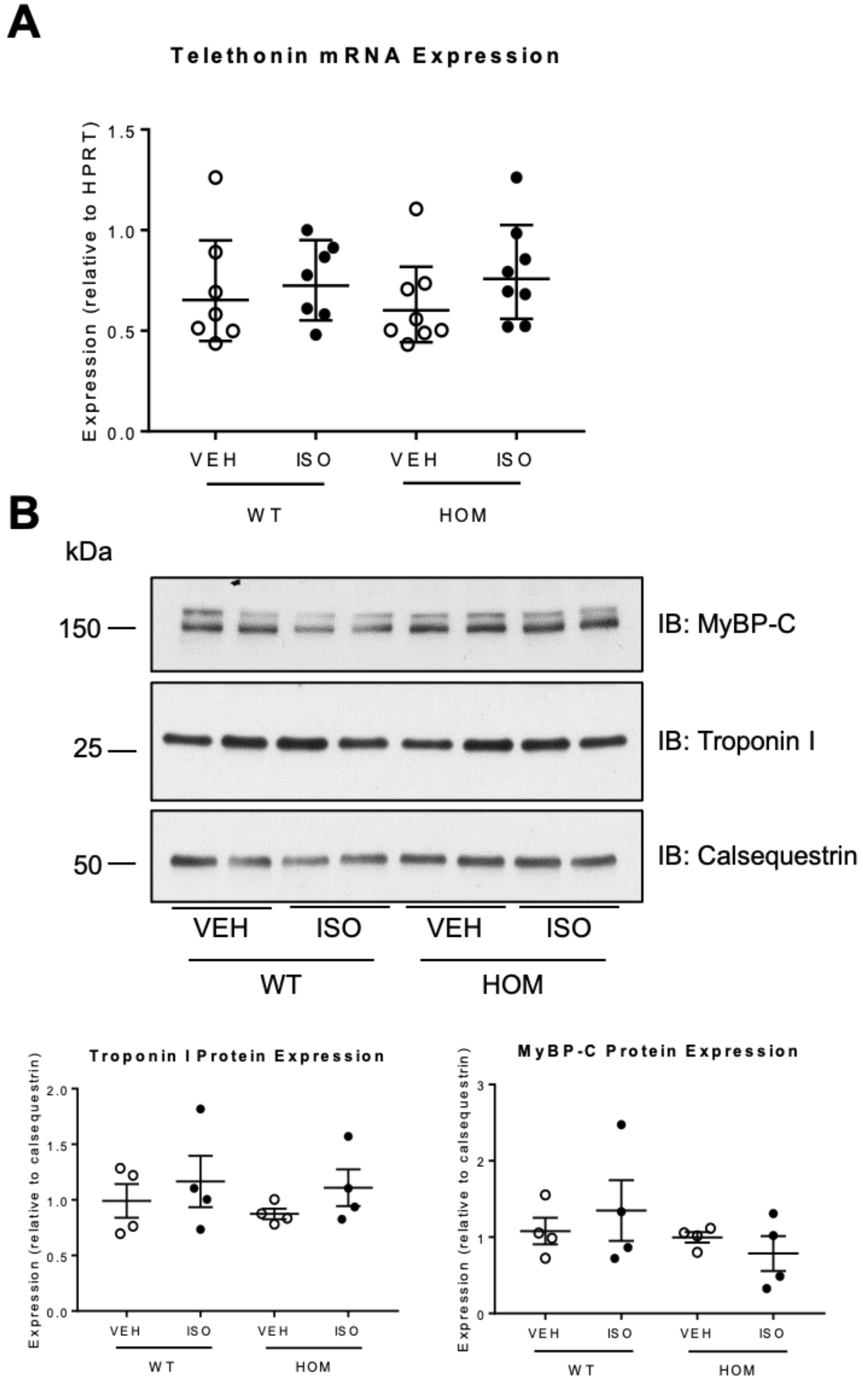

Supplementary Figure 7: Effects of sustained β-adrenergic stimulation on telethonin mRNA expression and sarcomeric protein expression in male *Tcap*$^{S157/161A}$ KI mice. **(A)** Real-time qPCR analysis of telethonin (*Tcap*) mRNA expression in cardiac tissue using the $2^{-\Delta\Delta CT}$ method (*Hprt* used as a reference gene) (n=7-8). **(B)** Immunoblot analysis of heart homogenates subjected to SDS-PAGE. Densitometric analysis of immunoblots (n=4), (lower panel). mRNA data expressed as geometric mean ± geometric SD, protein data expressed as mean ± SEM; 2-way ANOVA with Tukey's post hoc test.

2.2 Supplementary Tables

Supplementary Table 1: Primer sequences for quantitative-PCR. Forward (FOR) and reverse (REV) primer sequences were obtained using MGH-Primer Bank Website (https://pga.mgh.harvard.edu/primerbank/).

Primer	Sequence (5’ - 3’)
Tcap FOR	CTTCTGGGCTGAGTGGAAAG
Tcap REV	CTGGTACGGCAGCTGGTATT
Pnmt FOR	CAGACCTGAAGCACGCTACAG
Pnmt REV	TAGTTGTTGCGGAGATAGGCG
Col1a1 FOR	GCTCCTCTTAGGGGCCACT
Col1a1 REV	CCACGTCTCACCATTGGGG
Col3a1 FOR	CTGTAACATGGAAACTGGGGAAA
Col3a1 REV	CCATAGCTGAACTGAAAACCACC
p53 FOR	CTCTCCCCCGCAAAAGAAAAA
p53 REV	CGGAACATCTCGAAGCGTTTA
p21 REV	CCTGGTGATGTCCGACCTG
p21 FOR	CCATGAGCGCATCGCAATC
Hprt FOR	TCAGTCAACGGGGGACATAAA
Hprt REV	GGGGCTGTACTGCTTAACCAG
Gapdh FOR	AGGTCGGTGTGAACGGATTTG
Gapdh REV	GGGGTCGTTGATGGCAACA

Supplementary Table 2: Baseline phenotyping of male *Tcap*$^{S157/161A}$ KI mice. Gravimetric and echocardiographic data (during diastole (d) and systole (s)) from 16 week male mice (n=9-10). HW/BW: heart weight to body weight ratio; IVS: interventricular septum thickness; LVID: left ventricular internal diameter; LVPW: left ventricular posterior wall thickness; LVAW: left ventricular anterior wall thickness; LV Vol: left ventricular volume. Data expressed as mean ± SD, 1-way ANOVA with Tukey's post hoc test.

	**WT**	**HET**	**HOM**
HW/BW	4.47 ± 0.5	4.74 ± 0.4	4.79 ± 0.3
Atria weight (mg)	7.55 ± 2.6	5.92 ± 1.1	5.74 ± 1.4
IVS;d (mm)	0.73 ± 0.1	0.68 ± 0.1	0.74 ± 0.1
IVS;s (mm)	1.01 ± 0.1	0.92 ± 0.1	1.00 ± 0.1
LVID;d (mm)	4.11 ± 0.3	4.37 ± 0.3	4.23 ± 0.3
LVID;s (mm)	2.83 ± 0.3	3.14 ± 0.3	2.94 ± 0.4
LVPW;d (mm)	0.80 ± 0.1	0.70 ± 0.1	0.78 ± 0.1
LVPW;s (mm)	1.16 ± 0.1	1.11 ± 0.1	1.18 ± 0.1
LVAW;d (mm)	0.78 ± 0.1	0.68 ± 0.1	0.74 ± 0.1
LVAW;s (mm)	1.09 ± 0.1	0.99 ± 0.2	1.04 ± 0.1
LV Vol;d (µl)	75.22 ± 10.6	86.91 ± 12.4	80.51 ± 15.0
LV Vol;s (µl)	30.93 ± 8.2	39.93 ± 10.7	34.65 ± 12.4
Heart rate (bpm)	499.4 ± 30.4	508.30 ± 51.1	547.89 ± 57.9
Stroke Volume (µl)	44.29 ± 4.2	46.98 ± 7.3	45.87 ± 6.6

Supplementary Table 3: Cardiac response to sustained β-adrenergic stimulation in male *Tcap*$^{S157/161A}$ KI mice. Gravimetric and echocardiographic data from mice prior to osmotic pump implantation (Pre) and 14 days later (Post) (n=7-9). HW/BW: heart weight to body weight ratio; AW/TL: atria weight to tibia length ratio; IVS: interventricular septum thickness; LVID: left ventricular internal diameter; LVPW: left ventricular posterior wall thickness; LVAW: left ventricular anterior wall thickness; LV Vol: left ventricular volume in (d) diastole and (s) systole. Data expressed as mean ± SD, * or † $p \leq 0.05$; 2-way ANOVA with Tukey's post hoc test (genotype and treatment group) or Sidak's post hoc test (treatment response). (†) indicates significance when compared to baseline (Pre) and (*) indicates significance when compared to VEH control.

	**WT VEH**		**WT ISO**		**HOM VEH**		**HOM ISO**	
	Pre	*Post*	*Pre*	*Post*	*Pre*	*Post*	*Pre*	*Post*
HW/BW	-	4.94±0.4	-	5.01±0.4	-	4.55±0.4	-	5.20±0.3*
Lung weight wet/dry	-	3.99±0.1	-	4.09±0.1	-	3.97±0.1	-	4.21±0.1*
AW/TL	-	0.43±0.1	-	0.51±0.1	-	0.35±0.1	-	0.59±0.1*
IVS;d (mm)	0.70±0.10	0.66±0.08	0.68±0.11	0.68±0.13	0.7±0.07	0.71±0.09	0.66±0.09	0.76±0.05
IVS;s (mm)	0.98±0.12	0.92±0.11	0.94±0.14	0.97±0.18	1.00±0.14	1.00±0.14	0.98±0.16	1.04±0.11
LVID;d (mm)	4.24±0.24	4.41±0.31	4.12±0.24	4.33±0.20	4.19±0.42	4.26±0.20	4.12±0.22	4.40±0.24
LVID;s (mm)	3.05±0.32	3.18±0.37	2.94±0.21	3.12±0.23	2.91±0.40	3.08±0.24	2.84±0.22	3.31±0.30†
LVPW;d (mm)	0.73±0.07	0.76±0.14	0.74±0.10	0.87±0.09†	0.74±0.09	0.75±0.04	0.77±0.10	0.85±0.13
LVPW;s (mm)	1.05±0.15	1.13±0.10	1.12±0.14	1.27±0.11	1.13±0.12	1.14±0.13	1.16±0.10	1.19±0.15
LVAW;d (mm)	0.70±0.06	0.72±0.06	0.69±0.10	0.70±0.07	0.68±0.10	0.71±0.09	0.70±.0.14	0.74±0.09
LVAW;s (mm)	0.96±0.14	0.97±0.09	1.02±0.22	0.89±0.10	0.96±0.13	0.98±0.07	1.01±0.25	1.01±0.11

LV Vol;d (µl)	80.8±10.7	88.6±14.4	75.4±10.4	84.6±8.7	79.3±19.6	81.5±9.3	75.2±9.3	88.2±11.3
LV Vol;s (µl)	37.2±9.3	41.2±10.8	33.7±6.1	38.9±6.8	33.4±11.7	37.6±7.5	30.8±5.6	44.9±10.6†
Ejection Fraction (%)	54.3±7.8	54.2±6.3	55.1±7.0	54.2±5.1	58.6±5.4	54.1±5.4	59.3±3.7	49.4±6.8†
Stroke Volume (µl)	43.6±7.9	47.4±5.5	41.7±8.8	45.6±5.2	45.9±9.4	43.9±5.3	44.4±4.7	43.2±6.8
Heart rate (bpm)	519.0 ±75.8	503.1 ±29.9	517.3 ±44.6	643.1 ±16.8*†	535.6 ±41.5	519.6 ±56.8	531.1 ±42.8	633.3 ±18.3*†
Cardiac Output (ml/min)	22.3±3.1	23.7±2.0	21.6±4.8	29.3±3.3*†	24.3±3.2	23.0±4.9	23.5±2.2	27.3±4.0

Supplementary Table 4: Cardiac response to acute β-adrenergic stimulation in male *Tcap*$^{S157/161A}$ KI mice. Echocardiographic data from mice at baseline (BL) and following i.p. injections of VEH or isoprenaline (ISO) at a dose of 0.1 mg/kg body weight (n=8). IVS: interventricular septum thickness; LVID: left ventricular internal diameter; LVPW: left ventricular posterior wall thickness; LVAW: left ventricular anterior wall thickness; LV Vol: left ventricular volume in (d) diameter and (s) systole. Data expressed as mean ± SD, * or † $p \leq 0.05$; 2-way ANOVA with Tukey's post hoc test (treatment response) or Sidak's post hoc test (genotype response). (†) indicates significance when compared to baseline (Pre), (*) indicates significance when compared to VEH control.

	**WT BL**	**WT VEH**	**WT ISO**	**HOM BL**	**HOM VEH**	**HOM ISO**
IVS;d (mm)	0.63±0.05	0.62±0.05	0.80±0.09†*	0.63±0.05	0.61±0.08	0.79±0.09†*
IVS;s (mm)	0.88±0.05	0.87±0.08	1.22±0.12†*	0.89±0.07	0.88±0.09	1.16±0.14†*
LVID;d (mm)	4.22±0.42	4.19±0.45	3.42±0.25†*	4.18±0.35	4.11±0.30	3.39±0.33†*
LVID;s (mm)	3.14±0.45	3.09±0.44	1.87±0.43†*	3.22±0.42	3.15±0.39	2.11±0.44†*
LVPW;d (mm)	0.71±0.08	0.70±0.10	0.84±0.10†*	0.72±0.09	0.75±0.08	0.95±0.11†*
LVPW;s (mm)	1.04±0.12	1.04±0.10	1.44±0.13†*	0.99±0.07	0.99±0.11	1.37±0.17†*
LVAW;d (mm)	0.64±0.05	0.65±0.05	0.77±0.09†*	0.62±0.08	0.64±0.05	0.77±0.06†*
LVAW;s (mm)	0.83±0.10	0.93±0.12	1.12±0.11†*	0.88±0.10	0.90±0.09	1.16±0.05†*
LV Vol;d (µl)	80.4±18.2	79.5±19.7	48.6±8.4†*	78.7±15.6	75.3±12.5	47.7±11.6†*
LV Vol;s (µl)	40.5±13.6	38.9±12.8	11.8±6.3†*	42.6±13.5	40.4±11.7	15.9±9.3†*
Heart rate (bpm)	503.3±50.0	502.8±59.9	678.4±29.2†*	523.8±41.6	535.6±54.6	669.5±30.0†*

Stroke Volume (µl)	40.0±5.3	40.6±7.2	36.8±4.5	36.1±4.7	34.9±3.8	31.8±3.6†
Cardiac Output (ml/min)	20.1±3.4	20.2±3.5	25.0±3.6†*	18.9±2.9	18.7±2.6	21.3±2.3†*
